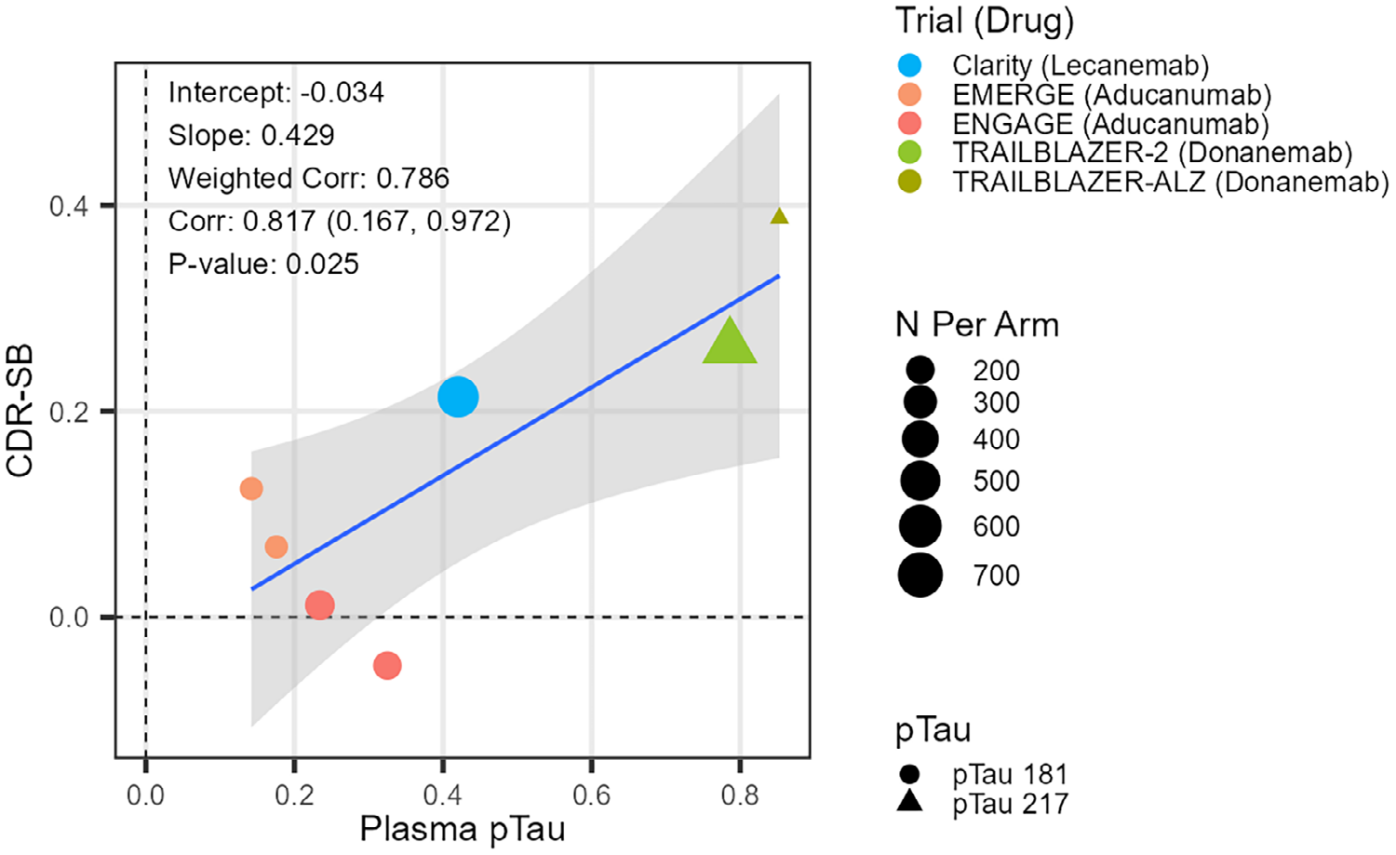# Leveraging recent advances in biomarkers to optimize early phase drug development in Alzheimer's Disease

**DOI:** 10.1002/alz70856_103841

**Published:** 2025-12-25

**Authors:** Garrett B Duncan, Tyler M Duke, Samuel B Johnson, Caleb W Dayley, Suzanne B. Hendrix, Craig Mallinckrodt, Samuel P. Dickson

**Affiliations:** ^1^ Pentara Corporation, Salt Lake City, UT, USA

## Abstract

**Background:**

Recent advances have been made in understanding the pathophysiology of Alzheimer's disease (AD) and the role played by biomarkers. Amyloid PET has been used for diagnosis and as the primary outcome to support accelerated approval in AD. However, amyloid PET is costly, time consuming, and equipment dependent. Therefore, we investigate the accuracy and sensitivity of plasma pTau as a convenient biomarker to predict clinical outcomes and disease progression in AD trials.

**Method:**

Published data from anti‐amyloid mAb studies were analyzed to assess the group‐level sample‐size weighted Pearson correlation between the treatment effects in plasma pT217, pT181 and clinical outcomes. The plasma pTau and clinical outcome correlation is compared to the corresponding correlation of amyloid PET with clinical outcomes in order to assess the relative merits of these biomarkers. To explore the potential of plasma pTau as a primary outcome, power calculation was performed to simulate proof‐of‐concept study designs on early drug development studies, assessing dose‐response and sample size.

**Result:**

We found that in the same studies in which amyloid PET was used to support accelerated approval applications, the group‐level correlations of plasma pT217, pT181 with clinical outcome group‐level effects were comparable to the corresponding correlations between amyloid PET and clinical outcomes. In addition, the Cohen's d effect sizes of plasma pT217 or pT181 as biomarker outcomes were greater than the Cohen's d values of clinical outcome assessments ADAS‐Cog, ADCS‐ADL, CDR‐SB, and their clinical composite, leading to higher power or lower sample sizes. Plasma pT217 or pT181 is 3‐fold more sensitive than clinical outcomes to measurement of disease progression, resulting in comparable power for one ninth of the sample size, since it is a squared relationship (see Figure).

**Conclusion:**

These plasma pTau biomarkers that are less costly and easier to obtain, may provide appropriate reliability as a predictor of clinical outcomes, similar to amyloid PET. Plasma pTau biomarkers also have a greater standard effect size than clinical outcomes and are more sensitive in predicting disease progression. Therefore, plasma pTau biomarkers could support early phase clinical trials with smaller sample sizes than clinical outcomes, using a more accessible and cost‐effective biomarker.